# Efficacy of anterior debridement and bone grafting with fusion using internal fixation combined with anti-tuberculosis chemotherapy in the treatment of subaxial cervical tuberculosis

**DOI:** 10.1186/s12893-022-01606-y

**Published:** 2022-04-27

**Authors:** Haopeng Luan, Kai Liu, Yao Wang, Alafate Kahaer, Weibin Sheng, Maierdan Maimaiti, Qiang Deng

**Affiliations:** 1 Department of Spine Surgery, The First Affiliated Hospital of Xinjiang Medical University, Ürümqi, 830054 Xinjiang China; 2 Department of Trauma and Microreconstructive Surgery, The First Affiliated Hospital of Xinjiang Medical University, Ürümqi, 830054 Xinjiang China

**Keywords:** Anterior surgery, Internal fixation, Subaxial cervical spine, Tuberculosis

## Abstract

**Background:**

To evaluate the efficacy of anterior debridement and bone grafting with fusion using internal fixation (BFIF) combined with anti-tuberculosis chemotherapy in the treatment of subaxial cervical spine tuberculosis (SCS-TB).

**Methods:**

Clinical and radiographic data of patients with SCS-TB treated by anterior debridement and BFIF at our hospital from January 2010 to December 2017 were analyzed retrospectively. The SCS sagittal parameters at the preoperative, postoperative, and final follow-up were documented and compared, including the Occiput-C2 angle, C2–C7 Cobb angle, local Cobb angle, spinal canal angle (SCA), C2–C7 sagittal vertical axis (C2–C7 SVA), the center of gravity of the head-C7 sagittal vertical axis (CGH-C7 SVA), T1 slope (T1S), neck tilt (NT), and thoracic inlet angle (TIA). The ASIA grade, NDI index, JOA score, and VAS score were utilized to assess the postoperative function recovery, and the complications were recorded.

**Results:**

A total of 23 patients were included in the study with a mean age of 46.74 ± 15.43 years, including 8 males and 15 females. All patients with SCS-TB were treated with anterior debridement and BFIF, with a mean postoperative follow-up time of 37.17 ± 12.26 months. The poisoning symptoms of TB were relieved in all patients, and ESR (42.09 ± 9.53 vs 8.04 ± 5.41, *P* < 0.05) and CRP (30.37 ± 16.02 vs 7.4 ± 2.68, *P* < 0.05) were decreased at the 3 postoperative months in the comparison of the preoperative. The C0–C2 Cobb angle, C2–C7 Cobb angle, local Cobb angle, SCA, TIS, C2–C7 SVA, and CGH-C7 SVA were corrected remarkably after surgery (*P* < 0.05). Further, there was a significant improvement in the JOA, VAS, and NDI with the comparison of the preoperative (*P* < 0.05).

**Conclusions:**

Anterior debridement and BFIF combined with anti-TB chemotherapy was a practical tool for the treatment of SCS-TB with the help of SCS sagittal parameters, which can remove the lesion completely, decompress the spinal cord compression, and correct the kyphotic deformity to restore the spine sagittal balance.

## Background

Spinal tuberculosis (TB) is the most common type of osteoarticular TB, accounting for 50% of all skeletal TB [1]. The incidence of cervical TB is rare, approximately 4.2–12% of spinal TB [2, 3]. However, the anatomical structure of the cervical vertebra is adjacent to the multi-level spinal nerve roots and central nerve structure, where lesions may easily result in the symptoms of spinal cord compression. Without timely anti-TB chemotherapy intervention, spine sagittal imbalance and kyphotic deformity may be caused by chronic vertebral destruction, which further aggravates spinal cord compression symptoms or even paraplegia [4–6].

Although anti-TB chemotherapy is still an effective and essential part of the treatment of skeletal TB, the surgical intervention also plays a non-negligible role in the treatment [1, 3, 7, 8]. Debridement and reconstruction surgery combined with anti-TB chemotherapy for the treatment of subaxial cervical spine tuberculosis (SCS-TB) is recommended by most studies, especially for patients with spine sagittal imbalance [9–12]. It has been reported that the anterior approach or combined anterior and posterior approach debridement surgery is a practical treatment for SCS-TB [1, 11, 13]. However, the surgical approach is still controversial because of the complex bony structure of the subaxial cervical spine [1–3, 12, 14]. And few studies evaluate the clinical outcomes of anterior debridement and bone grafting with fusion using internal fixation (BFIF) using the preoperative and postoperative vertebral radiographic sagittal parameters.

For these, the purpose of this study was to assess the outcomes of anterior debridement and BFIF combined with anti-tuberculosis chemotherapy in the management of SCS-TB, by the evaluation of vertebral radiographic sagittal parameters.

## Methods

### Study design

From January 2010 to December 2017, the medical records and radiographs were evaluated retrospectively of patients with SCS-TB treated by anterior debridement and BFIF, after receiving the written informed consent from participants and approval from the Ethics Committee of our hospital. Inclusion criteria are as follows: radiological indications of SCS-TB (bone destruction, narrowed intervertebral disc space, and para-spinal abscess); hematological and pathological examinations of TB (abscess sinus, marginal bone destruction, caseating granuloma); TB poisoning symptoms (low-grade fever, pain, night sweats, and neurological dysfunction); managed by anterior debridement and BFIF combined with anti-TB chemotherapy. Patients were excluded for incomplete medical records, poor compliance, other treatments were performed, or follow-up time less than 2 years.

The previous surgical and medical treatment, associated injury or diseases, the record of antibiotics utilization, and biopsy results were recorded. The inflammatory indicators were documented, including C-reactive protein (CRP), procalcitonin (PCT), erythrocyte sedimentation rate (ESR), and T-SPOT.

### Preoperative management

The surgery plan was drafted based on the anteroposterior and lateral radiographs, CT, and MRI films of the entire spine. Patients were treated with standardized anti-TB chemotherapy (isoniazid 300 mg, rifampicin 450 mg, ethambutol 1200 mg, and pyrazinamide 1500 mg) for at least 2 weeks preoperatively. Surgery was scheduled when the symptoms of TB poisoning were significantly controlled (ESR < 50 mm/h, and CRP < 30 mg/L). However, for patients with severe vertebral body and intervertebral disc destruction, or obvious symptoms of spinal cord compression, surgical treatment can be performed after the pain is relieved and inflammatory indicators decreased.

### Surgical technique

The patient was placed in the supine position. After the general anesthesia took effect, the cervical spine was hyperextended slightly by padding a cotton pad under both shoulders, and the surgical area was disinfected and draped routinely. After X-ray fluoroscopic, an anterior transverse incision was performed on the right side of the anterior neck. The skin and subcutaneous tissue were incised layer by layer to enter from the inner edge of the sternocleidomastoid muscle, and the surrounding tissues were separated bluntly using a periosteal elevator. More attention should be paid to protecting the trachea, carotid artery, and throat wall, and the trachea and esophagus were retracted inward carefully. Then the carotid sheath was retracted outwards, and the anterior fascia of the exposed vertebra was incised layer by layer to expose the anterior aspect of the vertebral body. The anterior cervical abscess, destroyed the vertebral body, the intervertebral disc was removed, and bone destruction in the vertebral body was observed. The worm-like necrotic tissue was removed by a curette, and the posterior longitudinal ligament was exposed by a distractor. After confirming that there was no compression of the spinal cord, the edge of the vertebral body was trimmed to expose the subchondral bone. The autologous iliac bone or allogeneic bone of the corresponding size was taken to embed the defect and fixed by an anterior cervical titanium plate. After the X-ray fluoroscopic of the internal fixation position was satisfactory, a large amount of 0.9% saline was applied for irrigation. The drainage was placed, and the incision was sutured layer by layer.

### Postoperative management

The drainage tube was removed when the fluid volume was < 50 ml/24 h. The X-ray was re-examined on the 5th postoperative day to observe the stability of internal fixation. Patients were encouraged to wear a neck rest and walk with the help of walking aid to prevent joint stiffness and pressure ulcers. After discharge, bone healing was evaluated by the X-ray, CT, and MRI at 1, 3, 6, and 12 postoperative months. The anti-TB chemotherapy described above was continued for at least 10 months. Simultaneously, ESR, CRP levels, and liver and kidney function were monitored every 6 weeks.

### Data collection and outcome evaluation

The clinical demographic data, operative time, amount of intra-operative bleeding, and follow-up time for each patient were recorded. ESR and CRP tests were used to monitor TB activity. The American Spinal Injury Association (ASIA) spinal cord injury grade, Japanese Orthopedic Association (JOA) cervical function score, neck disability index (NDI), and visual analogue scale (VAS) for pain were used to assess the patients’ functional improvement and the quality of life. Anteroposterior and lateral X-rays of the whole spine in the standing position were performed to assess the correction of the deformity by measuring vector parameters, as follows: Occiput-C2 angle, C2–C7 Cobb angle, local Cobb angle, spinal canal angle (SCA), C2–C7 sagittal vertical axis (C2–C7 SVA), the center of gravity of the head-C7 sagittal vertical axis (CGH-C7 SVA), T1 slope (T1S), neck tilt (NT), and thoracic inlet angle (TIA). The complications were recorded, including sinus formation, bone graft collapse, and vertebral bone destruction.

### Statistical analysis

Data were input into a Microsoft Excel spreadsheet (Redmond, WA, USA) and reported as mean ± standard deviation (SD), then analyzed by the SPSS 20.0 software package (Chicago, IL, USA). The normality of the data was analyzed by the Shapiro–Wilk test. Comparisons between the preoperative and the postoperative were conducted using the chi-square test or t-test. Statistical significance was *P* < 0.05.

## Results

**Fig. 1:**
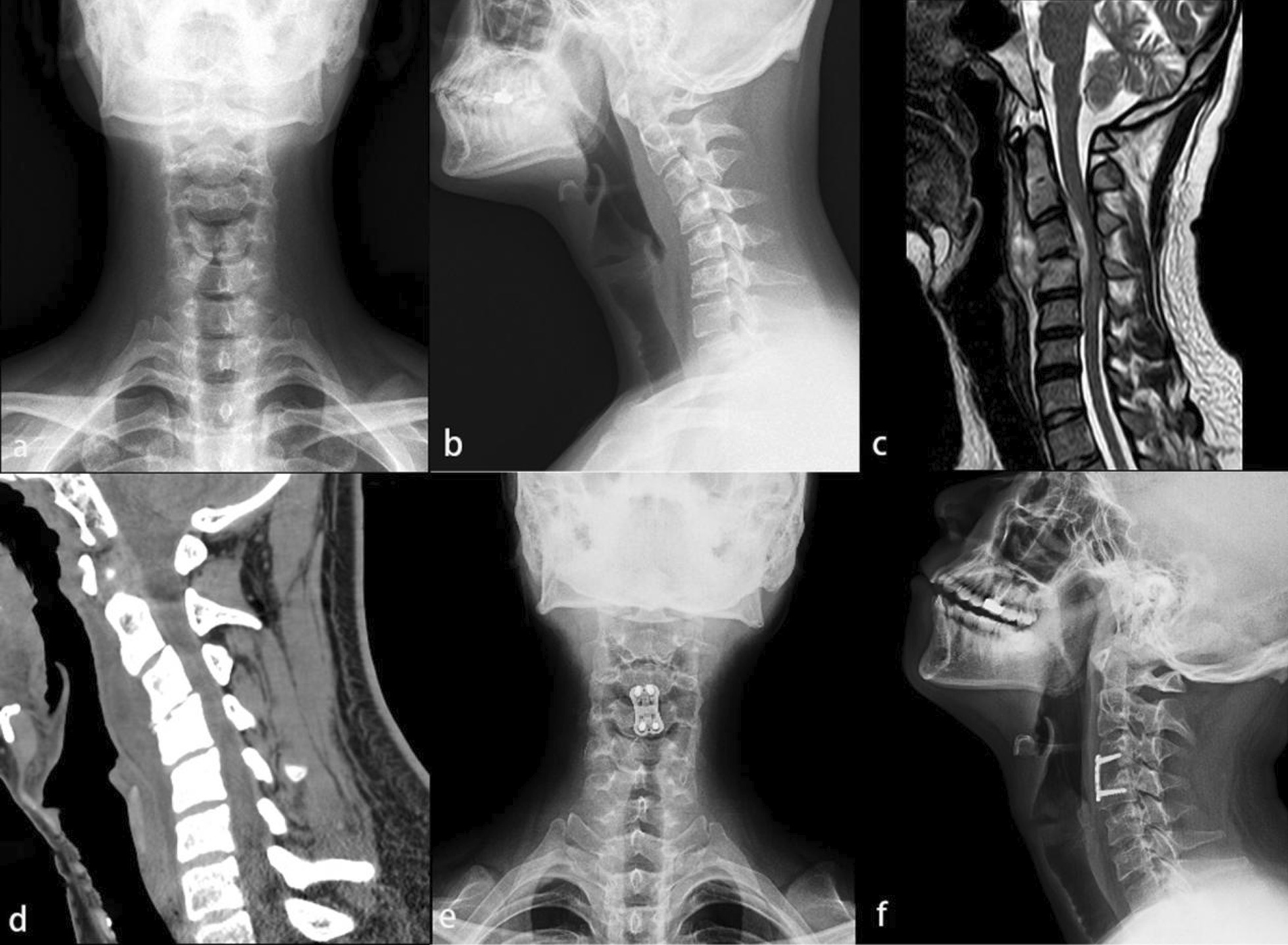
A 27-year-old woman with limited neck pain and mobility for 7 months and progressive limb weakness for 2 weeks. **a**, **b** Cervical anteroposterior and lateral X-ray showed C3, C4 vertebral destruction, C2–C4 local kyphosis. **c** Cervical MRI showed C3, C4 vertebral body partial loss, local kyphosis, and there was a significant abscess in front of the vertebral body. **d** Cervical CT showed C3 and C4 vertebral destruction. **e**, **f** Postoperative anteroposterior and lateral X-ray showed C3 and partial C4 vertebrectomy, the cervical sequence was corrected, and partial bone grafting fusion with stable internal fixation position

**Fig. 2:**
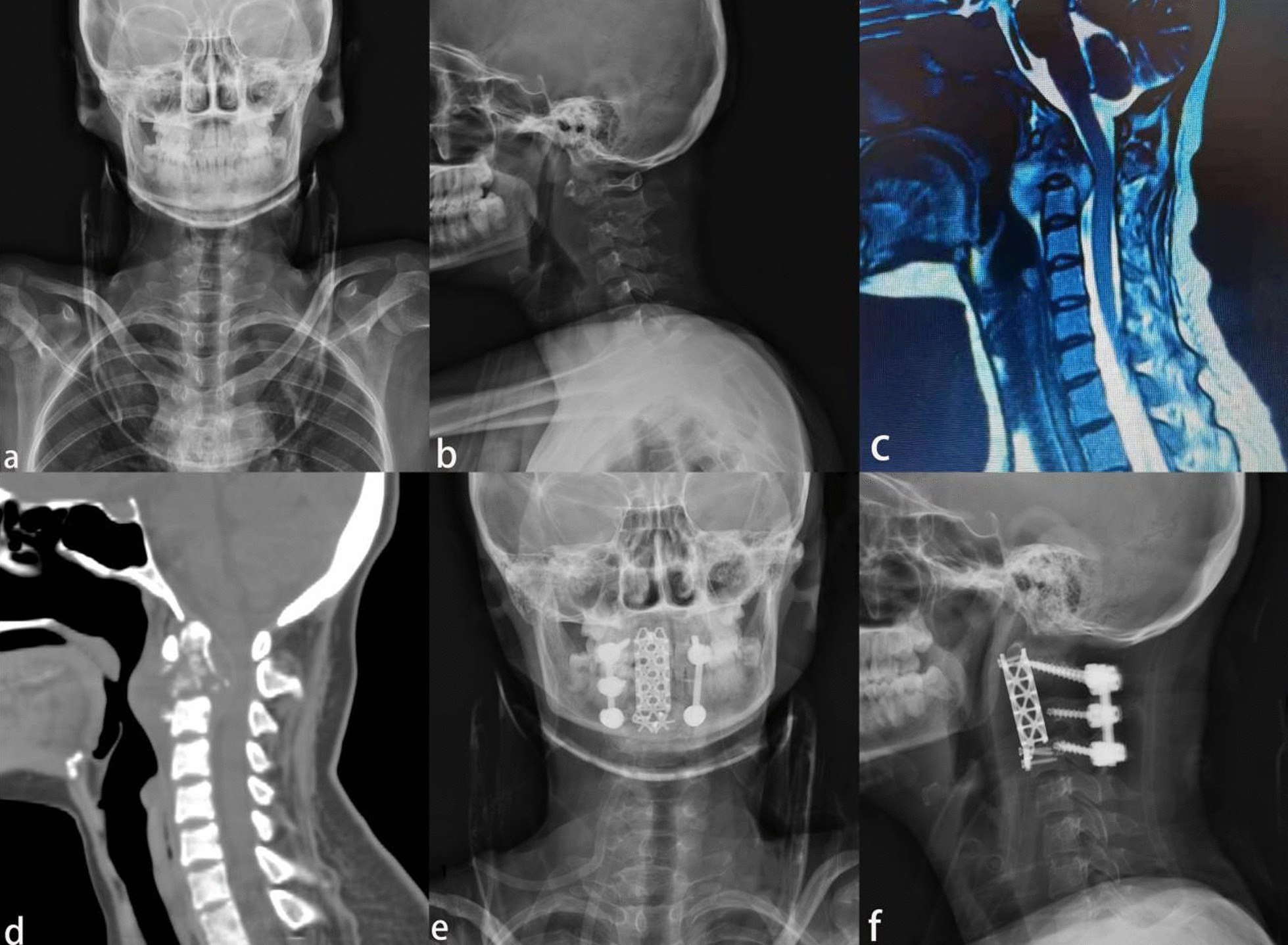
A 23-year-old woman with a 3-month of limited neck pain and mobility and 1 week of progressive weakness of the extremities. **a**, **b** Anteroposterior and lateral X-ray showed C1 and C2 vertebral destruction and local kyphosis from C1 to C3. **c** Cervical MRI showed significant loss of C2 vertebral body height, local kyphosis, compression of the sac, and localized prevertebral abscess. **d** Cervical CT showed C1 and C2 vertebral destruction, intraspinal encroachment, and local kyphosis from C1 to C3. **e**, **f** Postoperative anteroposterior and lateral X-ray showed C2 and partial C3 vertebrectomy, vertebral body reconstruction using titanium cage, and posterior approach surgery using cervical 2 pedicle screw and cervical 3, 4 lateral mass screw fixation

A total of 23 patients were included in the study with a mean age of 46.74 ± 15.43 years, including 8 males and 15 females. All patients with SCS-TB were treated with anterior debridement and BFIF (Figs. 1, 2), with a mean postoperative follow-up time of 37.17 ± 12.26 months. Neurological dysfunction occurred in 12 patients with a duration of 4–12 months, such as limited neck and shoulder movement, upper limb muscle weakness, and hypoesthesia. The postoperative inflammatory indicators of this cohort decreased significantly, which was summarized in Table 1. The poisoning symptoms of TB, such as low-grade fever, night sweats, weight loss, and fatigue were presented in 19 patients. At least 2–4 weeks of regular quadruple anti-TB chemotherapy (HREZ) before the operation were managed for all patients.

**Table 1:** Basic data of 23 patients with kyphotic cervical tuberculosis

Case	Age (years)/gender (M, F)	Lesion location	Follow-up time (months)	Approach of surgery	ESR (mm/h)		CRP (mg/L)	
					Preoperative	Three postoperative months^#^	Preoperative	Three postoperative months*
1	35/F	C6–C7	33	A	47	4	18.78	7.35
2	26/F	C3–C6	25	A	32	3	28.58	4.93
3	36/M	C6–C7	32	A	53	11	23.56	7.53
4	52/M	C3–C4	45	A	44	6	24.09	3.87
5	73/F	C5–C6	32	A	39	18	57.33	10.55
6	35/M	C6–C7	41	A	37	4	20.35	9.05
7	65/F	C3–C4	29	A	38	3	10.33	8.53
8	57/F	C2–C3	30	A	28	7	32.76	7.89
9	46/F	C3–C5	38	A	67	4	36.51	5.40
10	27/F	C3–C4	39	A	50	23	11.25	9.19
11	33/F	C4–C5	45	A	33	3	27.41	4.62
12	63/M	C3–C5	43	A	28	8	75.94	14.96
13	55/F	C6–C7	46	A	39	6	37.95	9.41
14	32/F	C4–C5	27	A	47	16	37.36	8.37
15	74/M	C2–C3	31	A	44	9	43.35	13.28
16	41/M	C5–C7	32	A	57	10	28.33	4.43
17	62/M	C3–C4	31	A	38	8	26.49	6.35
18	49/M	C5–C6	26	A	34	3	12.38	5.94
19	30/F	C4–C5	47	A	45	9	14.96	7.30
20	49/F	C5–C6	28	A	44	15	9.34	7.28
21	63/F	C4–C6	34	A	41	3	47.98	9.88
22	49/F	C5–C6	37	A	32	5	38.76	7.61
23	23/F	C1–C3	84	A	51	7	34.79	9.06

*A* anterior approach; *CRP* C-reactive protein; *ESR* erythrocyte sedimentation rate

*P < 0.05, comparison between the preoperative and 3 postoperative months

^#^*P* < 0.05, comparison between the preoperative and 3 postoperative months

The poisoning symptoms of TB were relieved in all patients, and ESR (42.09 ± 9.53 vs 8.04 ± 5.41, *P* < 0.05) and CRP (30.37 ± 16.02 vs 7.4 ± 2.68, *P* < 0.05) were decreased at the 3 postoperative months in the comparison of the preoperative (Table 1). The positive result of blood culture was observed in 17 patients (73.9%). The parameters of spine sagittal imbalance were effectively improved after surgery (Table 2). Specifically, the C0–C2 Cobb angle, C2–C7 Cobb angle, local Cobb angle, SCA, TIS, C2–C7 SVA, and CGH-C7 SVA were corrected remarkably (*P* < 0.05). Further, there was a significant improvement in the JOA, VAS, and NDI after surgery (*P* < 0.05, Table 3). According to the ASIA classification, incomplete spinal nerve injury below the C4 level was observed in 5 patients (paresthesia and muscle strength < grade 3). Lower extremity muscle strength was unable to completely counteract resistance observed in 4 patients. These spinal cord compression symptoms were improved after treatment, which recovered to grade E. There was no re-fracture or displacement of the internal fixation in all patients. The sagittal balance of the SCS was obtained and no recurrence of TB in this cohort.

**Table 2:** Comparison of sagittal parameters in the preoperative, postoperative, and the final follow-up

Parameter	Preoperative	Postoperative	Final follow-up	*P*^#^	P*
C0–C2 Cobb angle (°)	− 25.51 ± 7.17	− 20.80 ± 5.34	− 20.53 ± 2.89	0.015	0.017
C2–C7 Cobb angle (°)	14.26 ± 9.93	− 15.03 ± 8.10	− 14.81 ± 7.37	< 0.001	< 0.001
Local Cobb angle (°)	25.13 ± 8.28	− 9.74 ± 3.71	− 10.04 ± 3.54	< 0.001	0.002
Spino cranial angle (°)	91.74 ± 8.96	80.16 ± 7.05	81.65 ± 6.78	< 0.001	0.002
TI Slope (°)	15.04 ± 7.43	19.16 ± 8.36	19.42 ± 8.23	0.008	0.043
Neck tilt (°)	43.54 ± 9.89	40.38 ± 11.09	40.59 ± 10.74	0.041	0.016
Thoracic inlet angle (°)	58.62 ± 12.59	59.55 ± 9.46	60.01 ± 9.15	0.047	0.003
C2–C7 SVA (mm)	36.48 ± 10.35	11.65 ± 4.38	8.44 ± 3.01	< 0.001	< 0.001
CG-7 SVA (mm)	45.34 ± 13.46	27.20 ± 12.63	24.70 ± 12.17	< 0.001	0.017

P*: Comparison between the preoperative and the final follow-up

*P*^#^: Comparison between the preoperative and the postoperative

**Table 3:** Comparison of JOA score, VAS score, and NDI in the preoperative, postoperative and final follow-up

Variable	Preoperative	Postoperative	Final follow-up	*P*^#^	P*
JOA cervical function score	6.31 ± 2.03	14.96 ± 1.58	15.87 ± 0.87	< 0.001	< 0.001
Cervical VAS score	7.30 ± 1.52	3.01 ± 0.90	1.74 ± 0.61	< 0.001	0.002
NDI	32.61 ± 5.47	13.69 ± 2.88	8.83 ± 2.31	< 0.001	< 0.001

P*: Comparison between the preoperative and the final follow-up

*P*^#^: Comparison between the preoperative and the postoperative

Complications occurred in 2 patients. Briefly, the muscle strength of both upper limbs decreased from grade 5 to 3 in one patient, successfully treated by nerve electrical stimulation therapy and physical rehabilitation. Furthermore, incision sinus formation was observed in one patient at 3 postoperative months, who were managed for additional debridement surgery.

## Discussion

The purpose of this study was to evaluate the efficacy of anterior debridement and BFIF combined with anti-TB chemotherapy in the treatment of SCS-TB and analyze the changes in vertebral radiographic sagittal parameters. In this cohort, the affected area was mostly concentrated in the C3–C6, where patients with more than two vertebrae involved were usually associated with local kyphotic deformity. The spinal cord compression and spine sagittal imbalance were remarkably improved in all patients after anterior surgery combined with anti-TB chemotherapy, with effective control of TB poisoning symptoms. The longer the duration of TB, the higher incidence of spine sagittal imbalance. The standardized anti-TB chemotherapy was always necessary for the management of SCS-TB. The purpose of the surgery was to decompress the spinal cord compression, and restore the stability of the vertebral body to achieve the spine sagittal balance.

SCS-TB was a severe type of osteoarticular tuberculosis, which cannot be ignored in animal husbandry areas and developing regions since the increasing incidence recently [1, 3]. In the view of anatomy, the compression symptoms caused by cervical TB may be concealed by the wide cervical spinal canal. The joints of the lower cervical spine were flexible, which led to rapid progress once infection took place here. If the treatment was not timely, symptoms of spinal cord compression (paresthesia, paralysis) or kyphotic deformity might be caused by the lesions. Hence, the patients with SCS-TB mostly were not treated until the disease developed at the mid or advanced stage. Via previous studies [4, 9, 11, 15–18], surgical treatment combined with standard anti-TB chemotherapy was recommended to be managed the patients with the above symptoms. The purpose was to remove the lesion completely, relieve the compression of the spinal cord, correct kyphotic deformity, and reconstruct the height and physiological curvature of the cervical spine [1, 11]. Cervical tuberculosis lesions were usually located in the anterior and middle column of the vertebra. Although there were several surgical approaches (anterior, anterior combined with posterior, and endoscopy-assisted anterior), the anterior approach was still a practical method for surgery, since the simple structure of incision, convenient procedure of internal fixation, sufficient exposure of lesion, and less recurrence of infection [1, 2]. In this cohort, all patients with cervical tuberculosis had lesions concentrated in the lower cervical spine (C3–C7), 23 patients with lesions involving more than two vertebral bodies, and 12 patients with typical symptoms of spinal cord compression. And all patients were successfully treated by the anterior debridement, BFIF combined with anti-TB chemotherapy.

SCS-TB accounted for 3–5% of all spinal tuberculosis [3, 14]. The lesions here adjacent to the trachea and esophagus brought out the high incidence of symptoms such as hoarseness of inspiration, anorexia, and cervical lymphadenopathy. At present, atlanto-pivotal lesions were mostly treated by the posterior approach of surgery to rebuild the vertebral stability [4, 13, 19], while the SCS-TB was managed by the anterior approach of surgery since the high rate of kyphotic deformity [11, 20–22]. In addition, the importance of cervical sagittal balance in surgical decision-making and postoperative evaluation had been emphasized. A higher incidence of kyphotic deformity may occur in patients with C2–C7 cobb angle > 0° or C2–C7 SVA > 4 cm, and it was recommended to provide the anterior approach of debridement and reconstruction surgery for the above patients to acquire clearance of infection. In this study, 23 patients with a mean C2–C7 Cobb angle and C2–C7 SVA of 14.26 ± 9.93° and 36.48 ± 10.35 mm returned to − 14.81 ± 7.37° and 8.44 ± 3.01 mm (P < 0.001), respectively. In our experience, the anterior surgery was the simplest incision to acquire clear exposure to the lesion and thus yielded early patient recovery. And the abscess and lesions of the intervertebral disc area should be resolved first, and then the diseased vertebral body. The range of debridement should be performed until the fresh blood was scraped from the upper and lower vertebral bodies to avoid excessive destruction of the vertebral body resulting in spinal instability. Appropriate trimming of the bone defect facilitated the placement of the bone graft. The Caspar retractor screws were placed parallel to the middle of the upper and lower vertebral bodies adjacent to the diseased vertebral body, which was helpful for the exposure and removal of the lesions, correcting the cervical kyphosis, and restoring the physiological curvature.

The use of internal fixation in the management of infectious diseases is still controversial [1, 2, 14]. Although previous theories had suggested that biofilms form on the surface of internal fixation, which may be not conducive to the control of infection [23]. However, the current view is that surgical procedures combined with anti-TB chemotherapy can effectively eradicate the *Mycobacterium tuberculosis* on the surface of internal fixation [10]. Restoration surgery using internal fixation in the treatment of spinal infection diseases was recommended, such as titanium plate and titanium mesh cage. Koptan et al. [24] reported a prospective, nonrandomized multicenter study of 30 patients with cervical tuberculous spondylodiscitis was successfully treated by radical debridement and decompression using a titanium mesh cage. Besides, Mao et al. [22] presented a series of 21 patients with SCS-TB effectively managed by anterior debridement and bone grafting using internal plate fixation. As far as we were considering, the significance of bone graft was to form a bridge between the upper and lower vertebral bodies, and satisfactory fusion depended on the osteogenesis process with stable fixation. Immediate stabilization of the vertebral body could be provided by the titanium plate, which was able to restore cervical curvature and allow early rehabilitation. However, it is not always necessary to pursue a large amount of autogenous bone for bone grafting when applied to the titanium mesh cage, which can reduce the incidence of donor site complications (pain and poor wound healing). And the sharp dentate structure of the titanium mesh cage strengthened the shear resistance, which enhanced the torsional strength of the bone graft. Though, the application of titanium mesh cage was limited by the extent of vertebral lesions, which was suitable for involving more than three vertebral bodies. Although the previous studies illustrated the practical efficacy of titanium mesh cage, its application of it still needed to be decided on the extent of the lesion. In this study, there were nine patients managed by the titanium mesh cage and fourteen patients with a titanium plate. None of displacement, nonunion, or pseudoarthrosis formation took place.

It was also necessary to recognize that anti-TB chemotherapy was the fundamental part, surgical treatment was only an auxiliary tool [1–3, 8, 12]. The satisfactory result of surgery depended on the standardization and rational utilization of anti-TB drugs (early, combined, adequate, regular, and consistent) [8]. The recurrence of infection may be caused by ignorance of the role of anti-TB chemotherapy, which failed the entire treatment regimen. In our cohort, the 2HRZE/4HR regimen was conducted for patients and no recurrence of the infection. However, the side effects of anti-TB chemotherapy should be carefully observed during follow-up, and intervention should be applied timely, such as detection of liver and kidney function, psychological consult, etc.

The limitations of this study should also not be ignored. First of all, there was no mature treatment algorithm for the resolution of the SCS-TB. In addition, there was a lack of prospective series of SCS-TB treated with anti-TB chemotherapy, anterior or combined anterior and posterior approach surgery. Hence, a prospective study of more samples and multi-center is of more clinical significance.

## Conclusion

Patients with SCS-TB were successfully treated by anterior debridement and BFIF combined with anti-TB chemotherapy, which can remove the lesion completely, decompress the spinal cord compression, and correct the kyphotic deformity to restore the spine sagittal balance. The lesion level, vertebrae destruction, and degree of kyphosis deformity can be presented by the radiographic sagittal parameters, which were of great importance for surgical strategy.

## Data Availability

The data sets generated and analyzed during the current study are not publicly available due to restrictions on ethical approvals involving patient data and anonymity but can be obtained from the corresponding author on reasonable request.
